# Immune Checkpoint Inhibitor-Associated Acute Kidney Injury: A Single-Center Experience of Biopsy-Proven Cases

**DOI:** 10.3390/jcm14093231

**Published:** 2025-05-06

**Authors:** Andreas Kommer, Marco Stortz, Daniel Kraus, Julia Weinmann-Menke

**Affiliations:** Department of Internal Medicine I, University Medical Center of Johannes Gutenberg University, 55131 Mainz, Germany; marco.stortz@unimedizin-mainz.de (M.S.); d.kraus@uni-mainz.de (D.K.); julia.weinmann-menke@unimedizin-mainz.de (J.W.-M.)

**Keywords:** ICI-AKI, immune-related adverse events, onconephrology, checkpoint inhibitor

## Abstract

**Background**: Immune checkpoint inhibitor therapy (ICI) has greatly changed cancer therapy in recent years. The main side effects are immune-related adverse events (irAEs) that can affect any organ system. With the widespread use of ICIs, even rare irAEs, like acute kidney injury due to ICI-induced nephritis (ICI-AKI), have become a more common complication. **Methods**: All ICI-treated patients who underwent a kidney biopsy for AKI at a single academic center between January 2020 and December 2023 were analyzed. **Results**: We identified twelve cases of biopsy-proven ICI-AKI. The median follow up was 11.5 months. All cases showed acute interstitial nephritis (AIN) on the biopsy. Melanoma was the most common cancer, and dual-checkpoint inhibition with Ipilimumab and Nivolumab was the most common regimen. Extrarenal irAEs were present in only 25% of cases. Two-thirds had concomitant medication with proton pump inhibitors (PPIs). Only four patients completely recovered their kidney function, and one patient remained on kidney replacement therapy. **Conclusions**: AIN is a common cause of AKI in ICI-treated cancer patients. Although they respond well to steroid treatment, full restitution of kidney function occurs in less than half of the subjects. As ICIs are increasingly used in cancer management, more research on the prevention and treatment of ICI-associated AKI is needed.

## 1. Introduction

Immune checkpoint inhibitors (ICIs) have become a cornerstone for the management of many different types of cancers [1]. For the programmed cell death protein 1 (PD-1) target pathway alone, more than 30 different histotypes have been identified [2], with now around 40% of cancer patients in the United States being eligible for treatment with ICIs [3]. Checkpoint inhibitors work by blocking inhibiting pathways in T-cells, either via the transmembrane protein CTLA4 (Ipilimumab) or via PD-1 (Pembrolizumab, Nivolumab, Cemiplimab) or its ligand PD-L1 (Atezolizumab, Durvalumab, Avelumab). This way, T-cells are enabled to recognize and destroy cancer cells that previously evaded recognition via these pathways [1]. Apart from improving survival for these patients, ICI therapy has a better safety profile than conventional chemotherapy [4]. However, immune-related adverse events (irAEs) that can affect any organ system and mimic autoimmune disease are the most common side effects, leading to interruption or discontinuation of therapy. The severity of symptoms can range from mild to life threatening [5]. The most common complications affect the skin (9–34%) and gastrointestinal tract (6–45%) and are usually treated with topical or systemic corticosteroids. Other commonly affected organ systems include the lungs (1–5%), the endocrine system (7–38%), and the kidneys (1–5%) [5]. Of these acute kidney injury (AKI) due to ICI-induced nephritis (ICI-AKI) is a rare yet serious complication that prompts immediate attention [6]. The definition of ICI-AKI varies between studies but mostly can be summarized in meeting the Kidney Disease Improving Global Outcome (KDIGO) criteria for acute kidney injury and physicians attributing AKI to the use of ICIs. This indirectly includes excluding other causes of AKI like prerenal azotemia or sepsis, which are highly prevalent in this subset of patients. Traditional non-invasive tests, like urinalysis, have proven to be unspecific in the context of ICI-AKI. To avoid this problem in this study, we analyzed a cohort of ICI-treated patients who presented with AKI between January 2020 and December 2023 at our university medical center in Germany and subsequently underwent a kidney biopsy.

## 2. Materials and Methods

All patients undergoing a kidney biopsy at our center between January 2020 and December 2023 for acute kidney injury under ICI therapy were included in this study. Demographic characteristics, laboratory findings, medication, medical history, and biopsy results were obtained via manual chart review. Stages of AKI were defined as per the KDIGO guidelines. Baseline creatinine was defined as creatinine before the acute kidney injury event. Urine studies were obtained the day before the biopsy was performed. “Complete recovery” was defined as a creatinine value of less than 1.5 times the baseline creatinine. “Partial recovery” was defined as stable non-dialysis-dependent chronic kidney disease. “No recovery” was defined as dependency on kidney replacement therapy. The longest available time period for analysis as of December 2024 was used for analysis.

The kidney biopsy was performed percutaneously under ultrasound guidance. Histopathological interpretation included standard hematoxylin and eosin staining, as well as immunohistochemistry staining for IgA, IgG, IgM, C1q, and C3c.

Values are presented as median and interquartile range for continuous variables and as numbers and fractions in percentage for categorical variables. Due to the small sample size, comparisons across the groups “complete recovery” and “partial/no recovery” were evaluated using the Mann–Whitney U test for continuous variables and Fisher’s exact test for categorical variables. *P*-values of < 0.05 are considered statistically significant. Statistical analysis was performed using IBM SPSS Statistics for Windows, Version 29.0, Armonk, NY, USA: IBM Corp.

## 3. Results

Twelve subjects with ICI-associated AKI were included in the analysis, including eight women and four men. The median follow-up time was 11.5 months (9.13–27.5). The mean age was 64 years (35–78) (Table 1). The most common type of cancer was melanoma, with around 50% of cases. A total of 67% of patients were receiving dual-checkpoint inhibition with Ipilimumab and Nivolumab. The median duration of ICI therapy was 2.5 months, with one case of ICI-AKI occurring 31 months after the initiation of therapy. Extrarenal irAEs were uncommon and were found in only 25% of cases. This included two cases of hypophysitis and one case of hepatitis. Comedication with PPIs was found in 67% of cases. Hypertension was the most common preexisting condition. Since a biopsy is rarely performed for stage 1 AKI, the cohort only consists of stage 2 and stage 3 AKI, with the majority of patients suffering from stage 3 AKI. Two patients needed initiation of kidney replacement therapy, one of whom partially recovered kidney function after 7 days, and the other remained dependent on dialysis. Most patients partially recovered their kidney function with a sustained impaired kidney function. Only four patients (33%) completely recovered despite adequate steroid treatment in all but one case, and one remained on dialysis (Table 1). The patient who did not recover kidney function presented with oliguric kidney injury and massive peripheral edema with 14 kg of weight gain. Even after sufficient ultrafiltration with hemodialysis and high-dose diuretic therapy, no adequate diuresis was achieved, and an attempt to discontinue dialysis failed.

The subjects with only partial or no recovery differed from the subjects with complete recovery in several ways. PPI use was more common in those in the partial or no recovery groups, although it did not reach statistical significance. NSAID use was seen in one patient in the partial recovery group. A slightly higher creatinine at diagnosis and baseline was observed in those who only partially or did not recover their kidney function. This finding did also not reach statistical significance. Overall, no severe proteinuria or albuminuria was seen in any of the cases. All urine dipsticks showed a low specific gravity of <1.020. Urine microscopy was unspecific, with pyuria in seven of the twelve cases (58%) and hematuria in five of the twelve cases (41%) (Table 2). IFTA was not different between the groups. To our knowledge, a rechallenge occurred in only one case. Complete or partial recovery to stable creatinine occurred between two and twelve weeks, but it took up to six months in some cases. Varying doses of steroids were used in all but one case, with doses ranging from 100 to 250 mg of prednisone equivalent per day as an initial dose. Steroids were tapered within six to twelve weeks as per clinical improvement. The patient who did not receive steroids completely recovered kidney function by stopping ICI therapy alone.

AIN was the main histological diagnosis in all cases, with eosinophils being present in five cases and granulocytes being present in two cases (Table 3). Neither type of immune cell seemed to have an influence on the prognosis of AKI. The severity of AIN also did not seem to influence prognosis. None except one biopsy showed severe signs of chronic kidney damage, like more than 50% IFTA, or severe glomerulosclerosis. Interestingly, the patient with 70% IFTA completely recovered their kidney function, likely due to sampling bias. Arteriosclerosis was the most common additional finding. One patient had minimal mesangial IgA nephropathy, and one patient had necrotizing vasculitis of one artery; both were most likely not related to ICI therapy. The clinical significance of these findings remained unclear.

## 4. Discussion

We examined all patients under ICI therapy who underwent a kidney biopsy for AKI during a 3-year timespan. All patients showed AIN on the kidney biopsy, which is in line with the reported 90% of ICI-AKI cases [7]. Except for one case of minimal IgA nephropathy, we did not find glomerular lesions, like glomerulonephritis or podocytopathies, as have been described [8]. Due to the high prevalence of IgA nephropathy, we do believe this finding is by chance rather than caused by ICI therapy. One case showed necrotizing vasculitis of one artery on the biopsy, likely without clinical significance. As in most studies, a high percentage of our patients simultaneously received PPI therapy [7,9,10,11]. Urine studies were as unremarkable as previous studies have suggested, with very small degrees of proteinuria or albuminuria and around a 50% occurrence of hematuria or pyuria [7,12]. As has been shown for most cases of AIN, urine-specific gravity was low in all of our cases [13]. All but one patient was treated with guideline-recommended steroid therapy. The prompt use of steroid therapy led to kidney recovery in most cases. The overall recovery rate was high in eleven out of twelve cases (92%) but showed a high rate of sustained impaired kidney function in seven out of twelve cases (67%). This is in contrast to the previously reported 28.7–45% [7,14,15], leading to a significant rate of chronic kidney disease in patients after an episode of ICI-AKI. This is likely caused by selection bias. Most larger studies not only included biopsy-proven cases but cases of clinically suspected ICI-AKI, probably confounding the findings by including other entities. On the other hand, our study did not include patients with stage 1 AKI. Excluding these patients might in turn lead to higher rates of CKD. Comparably sized cohorts of single-center ICI-AKI patients also showed much higher rates of recovery but also included clinically suspected cases of ICI-AKI [16,17]. In contrast to these case series, PPIs were not discontinued in any of our cases, possibly maintaining AIN. Observational studies have found an association between ICI treatment and the development of CKD without the patients experiencing episodes of AKI [18]. Our data do not provide sufficient information on the reasons or possible mechanisms for these high rates of CKD. The role of ICI therapy in the development and progression of CKD should be subject to further investigation.

In summary, our study has several limitations: its small sample size and, therefore, lack of statistically significant findings, the relatively short observation period, the de facto systematic exclusion of stage 1 AKI, and non-homogenous treatment strategies. It merely can be hypothesis generating, pointing future research in the direction of the development of chronic kidney disease after ICI-AKI.

It is likely that due to concerns of a further worsening of kidney function, a rechallenge has only been performed in one of our cases. This shows that there is a need for clearly stated recommendations regarding rechallenge in these patients. Today, the American Society of Clinical Oncology (ASCO) guidelines recommend that all patients with stage 2 AKI who do not improve within days of steroid therapy and all patients with stage 3 AKI should not be re-exposed to ICI therapy [19]. These recommendations might deny patients with quickly resolving stage 3 AKI the opportunity to receive the best-suited oncological therapy for them. Due to the low rate of recurrence of ICI-AKI of around 16% [20], the Spanish Onco-nephrology Working Group and, most recently, the American Society of Onco-nephrology have proposed more individual and flexible criteria for rechallenge. These criteria take the cancer response to ICI therapy, the period of resolution of AKI and concomitant medications into account [21,22]. Rechallenging these patients with ICI therapy if it is mandated to control the underlying malignancy might be safe under certain circumstances. Still, the decision to do so should be made on a case-by-case basis with factors like concomitant PPI or NSAID use, time to recovery, or alternative therapeutic options influencing this decision. The data for the risk of recurrence of ICI-induced AIN in cases severe enough to qualify for kidney biopsy are very limited. Further guidance for nephrologists and oncologists is urgently needed.

With an incidence of around 20% of AKI in ICI-treated patients, an AKI is a common phenomenon, but only 10–25% of these are attributable to ICI therapy, making differentiation an important task for treating clinicians. The aim is to identify those who are likely to profit from the prompt initiation of steroid therapy and avoid unnecessary exposure. To aid this decision, clinical features, like the timing of onset or the occurrence of other irAEs, are not helpful. Traditional urine analysis is neither sensitive nor specific for the identification of ICI-AKI, highlighting the value of a kidney biopsy. For those with contraindications to a kidney biopsy, like systemic anticoagulation or a singular kidney, non-invasive tests are needed to spare these patients from unnecessary corticosteroid therapy and withholding of life-prolonging oncological therapy. As highlighted by others, the future evaluation of non-invasive tests in clinical trials is needed [20,21]. In a small study, urine retinol binding protein to the urine–creatinine ratio together with C-reactive protein has been proposed as a biomarker to distinguish ICI-AKI from other entities [23]. An analysis of multiple T-cell-associated cytokine tumor necrosis factor α (TNF-α), interferon-γ (INF), interleukin-2/-4/-6/-8/-9/-10 (IL), neutrophil gelatinase-associated lipocalin (NGAL), and kidney injury molecule-1 (KIM-1) in 14 patients with proven ICI-AKI showed urine IL-2, IL-10, and TNF-α to be significantly elevated compared to non-ICI AKI patients [24]. This validates urinary TNF-α as a discriminatory marker, which has previously been shown to improve the pre-biopsy differentiation of AIN from other causes of AKI [25]. In another cohort of 24 ICI-AKI cases, blood levels of soluble IL-2 receptor were significantly higher in ICI-AKI compared with ICI-treated controls without AKI and non-ICI AKI cases [26]. Recently, Moledina et al. have suggested urinary CXCL9, an INF-γ-induced chemokine, as a diagnostic marker for AIN [27], which has previously been shown to be able to discriminate between ICI-induced AIN and other causes of AKIs in ICI-treated patients [28].

As in our cohort, the most common finding on the biopsy is acute interstitial nephritis, potentially triggered by other drugs, like PPIs or NSAIDS, and exacerbated under ICI therapy or induced by loss of self-tolerance to renal antigens under checkpoint inhibition. The first might be caused by the stimulation of drug-specific T-cells causing typical hypersensitivity reactions [29,30,31]. The second might be caused by the overexpression of PD-L1 by tubular cells [32]. Most reported cases are stage 2 or 3 according to KDIGO criteria, maybe pointing to the underreporting of stage 1 AKI, which might be attributed to other causes and might resolve without steroid therapy. Future longitudinal analysis of patients undergoing ICI therapy with regular sampling of urine and serum specimens could provide a deeper insight into potential biomarkers and help identify high-risk subgroups for the development of ICI-AKI. Prospective studies are necessary to establish evidence-based diagnostic and therapeutic algorithms for ICI-AKI.

## 5. Conclusions

AIN is a common cause of AKI in ICI-treated cancer patients. Although responding well to steroid treatment, the complete recovery of kidney function might be lower than previously expected, as it occurs in less than half of subjects. Neither traditional urine analysis, the history of concomitant irAEs, nor the duration of ICI therapy are specific enough to aid in diagnosing ICI-induced AIN. This highlights the value of a kidney biopsy in these patients. As ICIs are increasingly used in cancer management, more research on the diagnosis, prevention, and treatment of ICI-associated AKI is needed.

## Figures and Tables

**Table 1:** Characteristics of patients. CKD: chronic kidney disease; ICI: immune checkpoint inhibitor; NSAID: non-steroidal anti-inflammatory drug; NSCLC: non-small cell lung cancer; PPI: proton pump inhibitor; RCC: renal cell carcinoma.

Variable	
n	12
Age (years)	66.5 (56.5–70.5)
Female	8 (67%)
Baseline creatinine (mg/dL)	0.88 (0.73–1.05)
Baseline eGFR (mL/min/1.73 m^2^)	77 (61–93)
Follow up (months)	11.5 (7.4–30.5)
Comorbidities	
Hypertension	8 (67%)
Diabetes	1 (8%)
Heart disease	3 (25%)
Previous CKD	1 (8%)
Cancer	
Melanoma	6 (50%)
NSCLC	2 (17%)
RCC	1 (8%)
Penile-Ca	1 (8%)
Merkel cell-Ca	1 (8%)
Cholangiocarcinoma	1 (8%)
ICI drug	
Ipilimumab + Nivolumab	8 (67%)
Pembrolizumab	1 (8%)
Nivolumab	1 (8%)
Durvalumab	2 (17%)
Duration of ICI therapy (months)	2.5 (1–6)
Extrarenal irAEs	
Yes	3 (25%)
No	9 (75%)
Comedication	
PPI	8 (67%)
NSAID	1 (8%)
AKI stage	
Stage 1	0 (0%)
Stage 2	1 (8%)
Stage 3	11 (92%)
*RRT*	
Yes	2 (17%)
No	10 (83%)
Outcomes	
Complete Recovery	4 (33%)
Partial Recovery	7 (58%)
No Recovery	1 (8%)

**Table 2:** Comparison of fully recovered and not fully recovered patients. ACR: albumin–creatinine ratio; CKD: chronic kidney disease; IFTA: interstitial fibrosis and tubular atrophy; NSAID: non-steroidal anti-inflammatory drug; NSCLC: non-small cell lung cancer; PCR: protein–creatinine ratio; RCC: renal cell carcinoma.

Variable	Complete Recovery	Partial/No Recovery	*p*-Value
N	4	8	
Age	58 (40–68.5)	67 (65–71)	0.214
Female	2 (50%)	6 (66%)	0.547
Comorbidities			
Hypertension	2 (50%)	6 (66%)	0.406
Diabetes	1 (25%)	0 (0%)	0.333
Cardiovascular disease	1 (25%)	3 (38%)	0.594
Previous CKD	0 (0%)	1 (13%)	1
Cancer			
Melanoma	2 (50%)	4 (50%)	
NSCLC	0 (0%)	2 (25%)	
RCC	0 (0%)	1 (13%)	
Penile carcinoma	0 (0%)	1 (13%)	
Merkel cell carcinoma	1 (25%)	0 (0%)	
Cholangiocarcinoma	1 (25%)	0 (0%)	
ICI drug			
Ipilimumab + Nivolumab	3 (75%)	5 (63%)	
Pembrolizumab	0 (0%)	1 (13%)	
Nivolumab	0 (0%)	1 (13%)	
Durvalumab	1 (25%)	1 (13%)	
Duration of ICI therapy (months)	4.5 (1–14)	2.5 (1–4)	0.808
Extrarenal irAEs	1 (25%)	3 (38%)	0.382
Comedication			
PPI	1 (25%)	7 (88%)	0.067
NSAID	0 (0%)	1 (13%)	
Clinical findings			
AKI stage 1	0 (0%)	0 (0%)	
AKI stage 2	1 (25%)	0 (0%)	
AKI stage 3	3 (75%)	8 (100%)	0.333
KRT	0 (0%)	2 (25%)	0.545
IFTA (%)	10.0 (2.5–55)	12.5 (10–23)	0.683
Baseline creatinine (mg/dL)	0.83 (0.73–0.95)	0.95 (0.73–1.23)	0.368
Baseline eGFR (mL/min/1.73 m^2^)	88 (67–108)	72 (55–85)	0.214
Creatinine at diagnosis (mg/dL)	4.4 (2.7–6.4)	5.7 (4.6–10.0)	0.570
CRP at diagnosis (mg/dL)	154 (61–182)	90 (52–150)	0.461
Urine studies			
PCR (g/g creatinine)	0.32 (0.27–0.41)	0.32 (0.19–0.4)	0.788
ACR (g/g creatinine)	0.14 (0.06–0.22)	0.13 (0.07–0.16)	0.683
Microhematuria	1 (25%)	4 (50%)	0.301
Pyuria	2 (50%)	5 (63%)	0.333

**Table 3:** Summary of histological findings on a kidney biopsy. CR: complete recovery; PR: partial recovery; NR: no recovery; ICI: immune checkpoint inhibitor; GS: glomerulosclerosis; IFTA: interstitial fibrosis and tubular atrophy; AIN: acute interstitial nephritis; Ipi: Ipilimumab; Nivo: Nivolumab; Pembro: Pembrolizumab; Durva: Durvalumab.

Biopsy No.	ICI	Recovery	IFTA	Glomeruli	GS	Main Histological Findings	Additional Findings	Severity of AIN
1	Ipi/Nivo	CR	70%	20	1/20	Interstitial nephritis, mainly lymphoplasma cells	Necrotizing vasculitis of a single artery	Mild to moderate
2	Pembro	NR	15%	19	5/19	Interstitial nephritis, mainly plasma cells and some granulocytes	Moderate arteriosclerosis	Mild to moderate
3	Ipi/Nivo	PR	60%	30	4/30	Interstitial nephritis, mainly lymphoplasma cells	Moderate arteriosclerosis	Moderate to severe
4	Ipi/Nivo	PR	10%	20	2/20	Interstitial nephritis, mainly lymphoplasma cells	Moderate arteriosclerosis	Mild
5	Nivo	PR	10%	37	6/37	Interstitial nephritis, mainly lymphoplasma cells and some eosinophils	Mild arteriosclerosis	Moderate to severe
6	Ipi/Nivo	PR	10%	28	0/28	Interstitial nephritis, mainly lymphoplasma cells and some eosinophils	Moderate arteriosclerosis	Moderate
7	Ipi/Nivo	CR	0%	37	0/37	Interstitial nephritis, mainly lymphoplasma cells	Minimal mesangial IgA nephropathy	Severe
8	Ipi/Nivo	CR	10%	23	3/23	Interstitial nephritis, mainly lymphoplasma cells and some eosinophils	Mild arteriosclerosis	Mild to moderate
9	Ipi/Nivo	PR	0%	13	1/13	Interstitial nephritis, mainly lymphoplasma cells and a few granulocytes	Mild arteriosclerosis	Severe
10	Durva	PR	15%	17	2/17	Interstitial nephritis, mainly lymphoplasma cells and some eosinophils	Moderate arteriosclerosis	Severe
11	Durva	CR	10%	23	6/23	Interstitial nephritis, mainly lymphoplasma cells	-	Severe
12	Ipi/Nivo	PR	25%	8	1/8	Interstitial nephritis, mainly lymphoplasma cells and some eosinophils	Moderate arteriosclerosis	Moderate

## Data Availability

Raw data are available from the corresponding author (A.K.) upon reasonable request.

## Abbreviations

The following abbreviations are used in this manuscript:

MDPI	Multidisciplinary Digital Publishing Institute
DOAJ	directory of open-access journals
TLA	three letter acronym
LD	linear dichroism
ACR	albumin–creatinine ratio
AIN	acute interstitial nephritis
AKI	acute kidney injury
ASCO	American Society of Clinical Oncology
CRP	C-reactive protein
eGFR	estimated glomerular filtration rate
GS	glomerulosclerosis
ICI	immune checkpoint inhibitor
IFTA	interstitial fibrosis and tubular atrophy
IL	interleukin
INF	interferon
irAE	immune-related adverse event
KIM-1	kidney injury molecule-1
KRT	kidney replacement therapy
NGAL	Neutrophil gelatinase-associated lipocalin
NSAID	non-steroidal anti-inflammatory drug
NSCLC	non-small cell lung cancer
PCR	protein–creatinine ratio
PD-1	programmed cell death protein 1
PPI	proton pump inhibitor
RCC	renal cell carcinoma
TNF-α	Tumor necrosis factor α
